# The full range of ophthalmological clinical manifestations in systemic lupus erythematosus

**DOI:** 10.3389/fopht.2022.1055766

**Published:** 2023-01-26

**Authors:** Nikita Kedia, Vincent Theillac, Manuel Paez-Escamilla, Chad Indermill, Denise S. Gallagher, Raphaël Adam, Anne Lise Qu-Knafo, Fatima Amari, Caroline Bottin, Géraldine Chotard, Violaine Caillaux, Maté Strého, Neila Sedira, Emmanuel Héron, Pierre-André Becherel, Bahram Bodaghi, Sarah Mrejen-Uretski, Alain-José Sahel, David Saadoun, Marie-Hélène Errera

**Affiliations:** ^1^ Department of Ophthalmology, University of Pittsburgh School of Medicine, Pittsburgh, PA, United States; ^2^ Department of Ophthalmology, Quinze-Vingts National Eye Hospital, Sorbonne-Universités-UPMC & DHU ViewMaintain, Paris, France; ^3^ Ophthalmology Department, Avicenne Hospital, Bobigny, Paris, France; ^4^ Ophthalmology Department, Lariboisière Hospital, Paris, France; ^5^ Ophthalmology Department, Centre Explore Vision, Paris & Rueil-Malmaison, Paris, France; ^6^ Department of Internal Medicine, Quinze-Vingts National Eye Hospital, Sorbonne-Universités-UPMC & DHU ViewMaintain, Paris, France; ^7^ Dermatology and Clinical Immunology Department, Hôpital Privé d’Antony, Antony, France; ^8^ Ophthalmology Department, Pitié Salpêtrière Hospital, Sorbonne-Universités-UPMC & DHU ViewMaintain, Paris, France; ^9^ Internal Medicine department, Pitié Salpêtrière Hospital, Sorbonne-Universités-UPMC, Paris, France

**Keywords:** lupus, uveitis, uveitis (MeSH), posterior scleritis, idiopathic intracranial hypertension, ocular lupus, Lupus retinopathy, optic neuritis

## Abstract

**Purpose:**

To determine the full range of ophthalmological clinical manifestations in systemic lupus erythematosus (SLE) and to compare the systemic features associated with them.

**Methods:**

Files of 13 patients with ocular SLE (*n =* 20 eyes) diagnosed as per the American College of Rheumatology (ACR) 2012 revised criteria were retrospectively reviewed.

**Results:**

The following clinical manifestations were found: keratoconjunctivitis sicca (*n =* three patients), anterior uveitis associated with an inflammatory pseudo-tumor orbital mass (*n =* one patient, one eye), episcleritis and periorbital edema (*n =* one patient, two eyes), posterior scleritis (*n =* one patient, two eyes), bilateral papillary edema in the context of idiopathic intracranial hypertension (*n =* one patient, one eye), inflammatory optic neuritis (*n =* one patient, one eye), and lupus retinopathies with varying degrees of capillary occlusions mainly arteriolar (*n =* seven patients, 13 eyes) and larger arteries or veins (retinal arteries occlusions and retinal veins occlusions) (*n =* one patient, two eyes). Some patients presented with combined ophthalmological manifestations.

Systemic SLE was discovered by its ophthalmic manifestation in three cases (23%) and was previously known in the other 10 cases (77%). On average, ocular symptoms were seen 8 years after the initial diagnosis of SLE. Other systemic SLE disorders included cutaneous disorders (77%), joint disorders (38%), central nervous system (CNS) disorders (23%), renal disorders (38%), and oral ulcers (23%).

Treatment of the ophthalmic system manifestations of lupus included local steroid therapies along with systemic immunosuppression.

The most common laboratory ACR criteria were: high levels of antinuclear antibodies (ANA) (100%), positive anti-Sm (64%), anti-dsDNA (27%), low complement levels (27%), and positive antiphospholipid (APL) antibodies (18%).

**Discussion:**

SLE activity in the ophthalmic system is characterized by its functional severity and the range of involvement can be categorized by anatomical involvement: presence of anterior uveitis, episcleritis, scleritis, periorbital edema, posterior uveitis with retinal vascular ischemia, or papillary edema. Not currently part of the diagnosis criteria of the SLE ACR given its rarity, the ocular localization of the pathology led to the diagnosis of SLE in three cases; thus, developing a greater understanding of ocular lupus may help in identifying and treating systemic manifestations of lupus earlier.

## 1 Introduction

Systemic lupus erythematosus (SLE) is a heterogeneous condition of immunological origin. The occurrence of SLE might be influenced by genetic factors, sex, prolonged exposure to a particular environment, and random events. Defective immune regulatory mechanisms can lead to T- and B-cell hyperactivity and the production of pathogenic antibodies to components of the cell nucleus. This leads to widespread inflammation affecting many organ systems. Understanding of the pathophysiology of lupus remains incomplete, especially with regards to ocular manifestations of lupus.

Known ophthalmic disorders associated with SLE are dry keratoconjunctivitis (1), acute anterior uveitis (2), chronic blepharitis, scleritis (3), periorbital edema (rare) (4), and orbital mass syndromes (5) that can be complicated by ocular ischemia (6). The other structures of the anterior and posterior segments that may be affected by the ocular SLE are most often the retina, the cornea (7), the conjunctiva (8), and the episclera (9), along with the choroid, retinal vessels, and optic nerve. Retinal involvement appears to be a key feature of disease activity (10).

Here we describe a wide range of examples of SLE-related ophthalmic system involvement. We compare the ophthalmic findings in the patients with ocular SLE seen in our tertiary centers to the few ophthalmic manifestations taken into account by the British Isles Lupus Assessment Group (BILAG) (11). BILAG is a disease activity index for SLE in which 13 ophthalmological problems are taken into account (11): orbital inflammation/myositis/proptosis; keratitis, severe; keratitis, mild; anterior uveitis; posterior uveitis/retinal vasculitis, severe; posterior uveitis/retinal vasculitis, mild; episcleritis; scleritis, severe; scleritis, mild; retinal/choroidal vaso-occlusive disease; isolated cotton-wool spots (cystoid bodies); optic neuritis; and anterior ischemic optic neuropathy (11).

## 2 Methods

We have performed a retrospective evaluation of nine patients with ocular manifestations of SLE, seen between 1993–2018 in three tertiary reference centers for uveitis in France, and we have added four patients seen in 2021–2022 from the UPMC Eye Center, Pittsburgh, USA, to complete the full range of reported ocular SLE manifestations.

The diagnosis of SLE was made according to the American College of Rheumatology (ACR) criteria (12), with an assessment in the internal medicine (IM) or rheumatology department for each patient. The presence of anti-nuclear antibodies (ANA) was constant.

We retrospectively considered the following data: demographic data, date of SLE diagnosis, history of extraocular manifestations related to SLE, ophthalmic disease caused by SLE, and systemic treatments.

We examined the results of the slit lamp and fundus examinations, the measurement of the best corrected visual acuity (BCVA), and intraocular pressure. For retinal involvement, we used the following retinal imaging modalities results: color retinal fundus imaging of 50° field of view or 140° wide-fields, retinal fluorescein angiography (FA), indocyanine angiography (ICG), fundus autofluorescence (AF), and spectral-domain optic coherence angiography (SD-OCT).

## 3 Results

Thirteen adult patients who fulfilled ACR criteria (12) for SLE were seen in uveitis clinics for ophthalmic system manifestations of SLE (*n =* 20 eyes affected) at specialized ophthalmological hospital centers. Mean age at ophthalmic involvement was 47 years (range: 20–77 years), and all 13 patients were female (100%). On average, ocular symptoms occurred 8 years after the diagnosis of SLE (range: 0–20 years). Five patients (38%) presented with ocular symptoms within 1 year of being diagnosed with systemic lupus, including three patients (23%) whose systemic lupus was diagnosed based on its ophthalmic manifestations.

The most prevalent ophthalmological clinical features were lupus retinopathy with varying degrees of severity which affected eight patients (*n =* 15 eyes): one patient with large vessel occlusion, four patients with lupus retinopathy and severe vaso-occlusive diseases, and three patients with proliferative retinopathy. The vessels occlusions were mainly arteriolar (*n =* 13 eyes), but one case involved larger arteries or veins (retinal arteries occlusions and retinal veins occlusions) (*n =* two eyes). Sicca syndrome was present in three patients (*n =* six eyes). Few other ophthalmological disorders were present, such as one patient (*n =* one eye) presenting with anterior uveitis and inflammatory pseudo-tumor orbital mass, one patient presenting with episcleritis and periorbital edema (*n =* two eyes), one patient presenting with papillary edema revealing idiopathic intracranial hypertension (*n =* one eye), one patient with a posterior scleritis (*n =* two eyes), and one patient with inflammatory optic neuritis (*n =* one eye). Clinical presentations were very heterogeneous, as were visual acuity recoveries.

Systemic manifestations of SLE from the ACR criteria were as follows: cutaneous lupus was seen in 10 patients (77%), joint involvement secondary to lupus (arthritis) in six patients (46%), neurologic manifestations in three patients (23%), renal involvement in five patients (38%), and oral ulcers in three patients (23%).

Owing to the retrospective nature of the current study, immunological criteria for SLE were missing in two patients’ notes but were available in the remaining 11 cases. The most common laboratory ARC criteria (13) were as follows: antinuclear antibodies (ANA), the levels of which were outside laboratory range in all 11 cases (100%); followed by positive anti-Sm levels in seven cases (64%); positive anti-SSA levels in five cases (45%), anti-dsDNA levels, which were above the laboratory reference range in three cases (27%); anti-RNP levels, which were above the laboratory reference range in three cases (27%); low complement levels in three cases (27%); and positive anti-nucleosome antibody levels, in one case (9%). Antiphospholipid (APL) antibodies were present in two cases (18%). These patients had positive lupus anticoagulant (LA) antibodies but negative anticardiolipin (aCL) antibodies and negative anti-beta-2-glucoprotein-I antibodies. The level of LA antibodies was not reported in the two patients’ notes.

At the patients’ initial visits to ophthalmology, best corrected visual acuity (BCVA) in the affected eyes ranged from light perception (LP) to 20/20. A total of 10 patients (83%) had bilateral involvement (*n =* 20 eyes affected with ocular lupus). For nine eyes (45%), visual acuity was ≤ 20/63. Among these nine eyes, five (25%) showed improved BCVA after treatment. There were also three eyes (15%) that showed worsened BCVA during/after treatment.

Treatments varied depending on the specific ophthalmic symptoms and systemic manifestations of SLE. Multiple therapies were used for each of the 13 patients in the study. Corticosteroids were commonly used and were administered either intravenously (*n =* 8 patients), orally (*n =* 7), topically (*n =* 2), or through intraocular/periocular injections (*n =* 2). Other systemic immunosuppressive agents were also used to treat SLE, such as methotrexate (*n =* 2), mycophenolate mofetil (MMF) (*n =* 5), cyclophosphamide (CYC) (*n =* 5), and azathioprine (AZA) (*n =* 3). Hydroxychloroquine (HCQ) was also noted as a part of a treatment for SLE (*n =* 5). Monoclonal antibodies were used in some cases: belimumab (*n =* 1) and rituximab (*n =* 3). Ocular manifestations were treated with laser photocoagulation (*n =* 7), therapeutic pars plana vitrectomy with retinal delamination (*n =* 1), oral acetazolamide (*n =* 1), or intravitreal anti-VEGF injections (*n =* 3).

## 4 Case series

### 4.1 Anterior uveitis

#### 4.1.1 Case number 1

A 61-year-old female who presented to the ophthalmology department with blurred vision and associated redness was diagnosed with anterior uveitis in the right eye. Nine years prior to this, she had been diagnosed with discoid lupus erythematous (DLE) which presented as scalp lesions. She had an elevated ANA titers of 1/280 that normalized 1 year later along with persistent hypocomplementemia (low C4) and positive SSA and SM antibodies. She had been on long-term daily HCQ monotherapy. Upon diagnosis of anterior uveitis, the patient was treated with a topical steroid prednisolone acetate 1% taper, followed by topical difluprednate, because of persisting anterior segment inflammation, which led to resolution of uveitis for 2 years. The patient then had recurrent flares of anterior uveitis and anterior diffuse scleritis over the following 2 years. Each uveitis flare was treated with topical steroids or subconjunctival injections of dexamethasone at a dose of 4 mg, along with intraocular pressure-lowering medications. She also presented acutely with diplopia and was diagnosed with optic perineuritis caused by an apical orbital pseudotumor. She was treated with a 5-day course of IV methylprednisone and was discharged with a prescription for oral prednisone (60 mg per day followed by a taper) and long-term oral methotrexate (15 mg per week) for maintenance, the later discontinued because of side effects. Following an episode of bilateral scleritis, the patient started a course of oral MMF (1500 mg per day) for both the ophthalmic and skin manifestations of lupus.

### 4.2 Episcleritis and periorbital edema

#### 4.2.1 Case number 2

**Figure 1:**
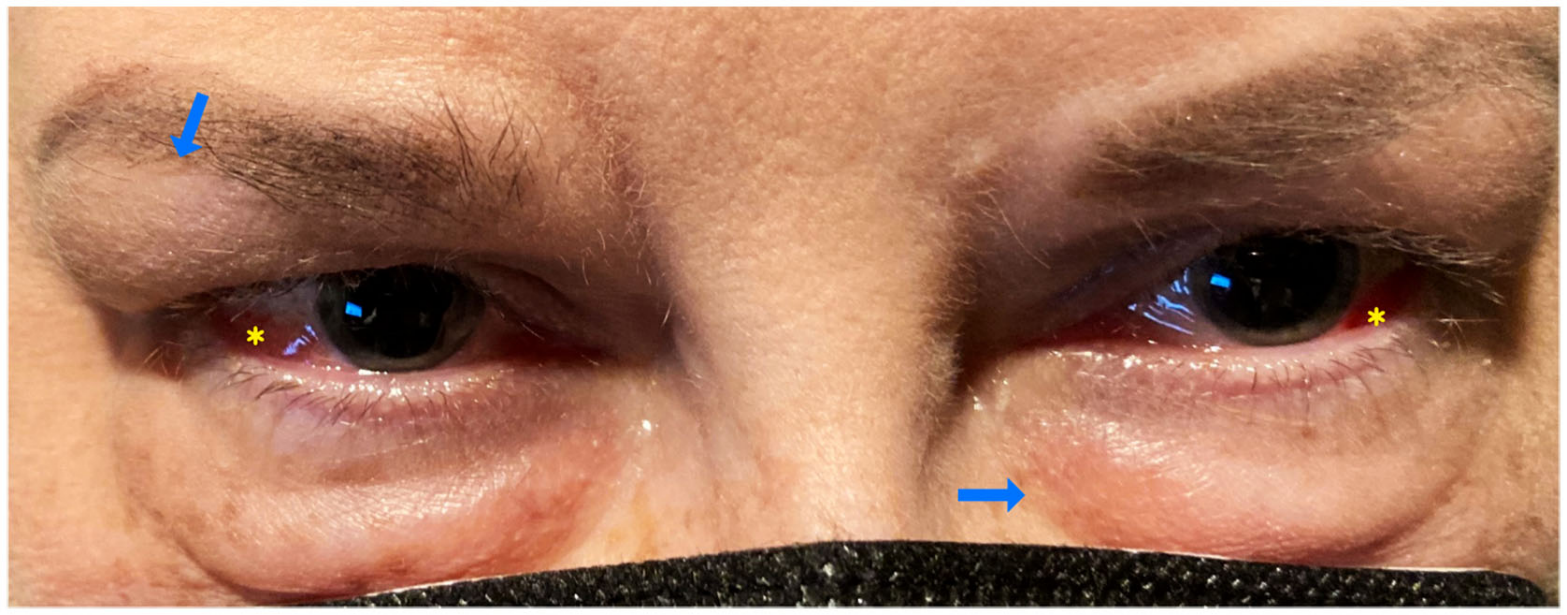
Case number 2. External photograph of a 56-year old-female with a medical history of SLE and Sjogren disease that consulted for keratoconjunctivitis sicca, she also complained of polyarthralgias fatigue, oral ulcers and photosensitivity. Ophthalmic exam showed bilateral periorbital edema (blue arrows), and diffuse anterior scleritis (yellow asterisks). At the time of eye presentation, she had been on oral MTX 15 mg weekly combined with 13 mgs of prednisone and belimumab infusions since the past two years.

A 56-year-old female patient with a 14-year past medical history of SLE, Crohn’s disease, Celiac disease, and Sjögren’s syndrome was found to have episcleritis and periorbital edema (**Figure 1**). SLE was diagnosed on positive ANA, polyarthralgias, fatigue, photosensitivity, oral ulcers, Sicca syndrome, Raynaud’s syndrome, pleurisy, pericarditis, and hypocomplementemia (low C3). She also had small fiber neuropathy/autonomic dysfunction with gastroparesis. Her laboratory test results showed positive rheumatoid factor, positive APL (positive lupus anticoagulant in laboratory work performed 15 years ago), positive Factor V Leiden, negative Sjögren’s antibody, negative SSA and SSB, negative anti-SM/RNP antibody, negative SM antibodies, negative anti-DNA antibody, and a normal, full blood count. She had been on a course of oral AZA with a dose of 150 mg daily that was switched to oral methotrexate (15 mg weekly) because the oral AZA led to the patient contracting a urinary tract infection and kidney stones. She was also on long-term prednisone (13 mg per day) and had been on steroids for the last 10 years. The lowest dose she had tapered to was 7–8 mg/day, but this could lead to flares in either her SLE and/or migraines. She had also been started on a belimumab infusion 2 years prior.

### 4.3 Large vessels occlusions: Branch retinal veins and arterioles occlusions

#### 4.3.1 Case number 3

**Figure 2:**
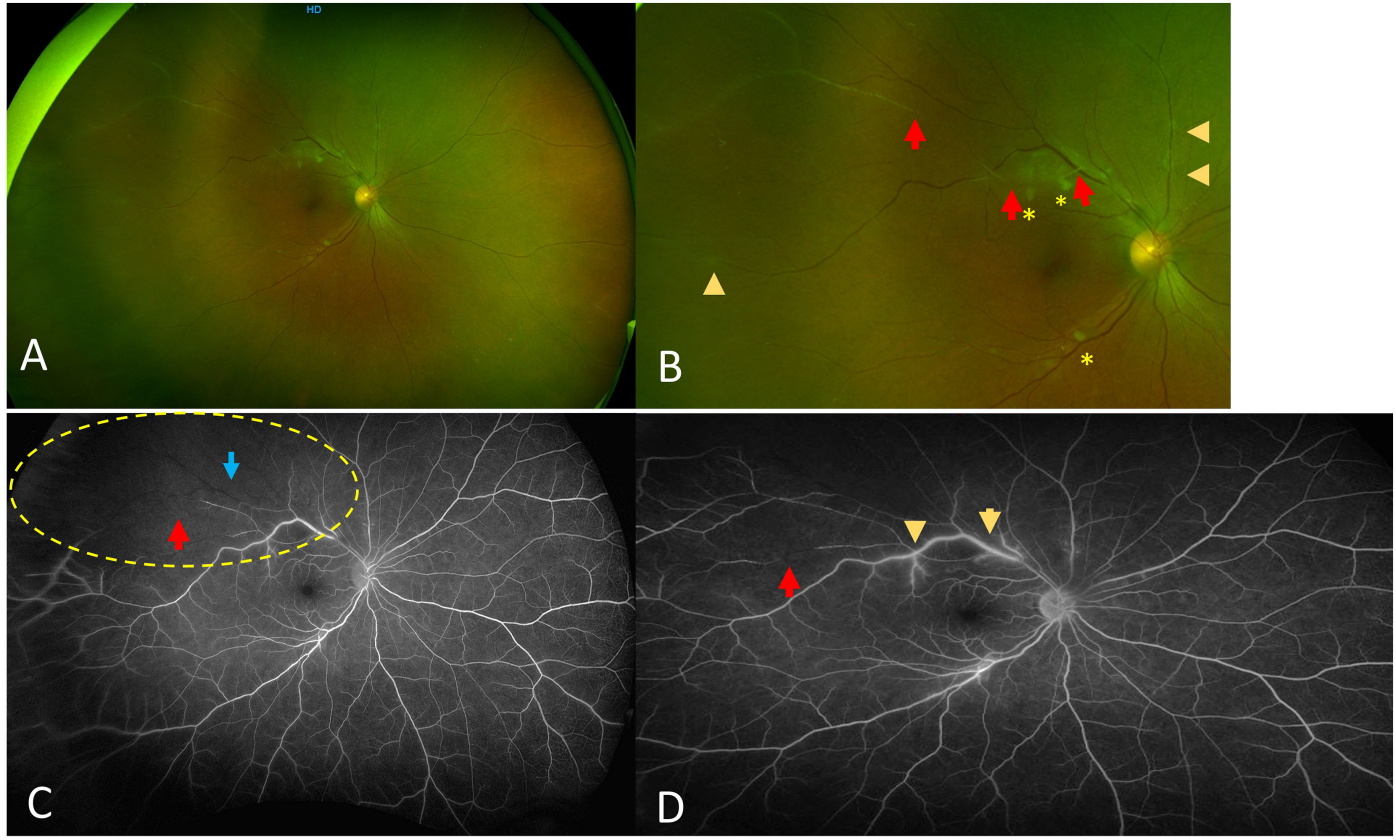
Case number 3. Optos fundus photos and wide field fluorescein angiography of a 61-year-old female with combined BRVO and BRAO due to mixed connective polyautoimmune SLE negative for antibodies for antiphospholipid syndrome but positive anti-RNP and meningo-encephalitis. **(A)** Wide view shows absence of vitritis with sheathing of both veins and arterioles. **(B)** close up that clearly shows sheathing from both veins (arrowheads) and arterioles (red arrows), asterisks show both veins and arterioles that are totally occluded with cotton-wool spots. **(C)** Intermediate phase FA shows sectoral lack of perfusion superotemporally (yellow dotted circle), with both arteriole (red arrow) and vein (blue arrow) showing lack of flow. **(D)** Late phase FA that shows staining of the veins compatible with periphlebitis (arrowhead) and partially occluded arteriolar flow proximally (yellow arrow) and distally (red arrow). The patient was treated with sectoral PRP following a period of eye quiescence.

A 61-year-old female patient presented with a combined branch retinal vein occlusion (BRVO) and a branch retinal arteriolar occlusion (BRAO) in the right eye, located at the temporal vascular arcade, and associated with retinal periphlebitis in both eyes (**Figure 2**). This patient, at the time of ophthalmic diagnosis, was suffering from SLE without antiphospholipid (APL) syndrome, along with sarcoidosis, diagnosed through a skin biopsy. The diagnosis of SLE was diagnosed after a test to determine the levels of by positive anti-dsDNA antibodies and positive anti-RNP antibodies. A lumbar puncture was performed during an episode of confusion and was suggestive of meningo-encephalitis secondary to SLE. Systemic treatment included a bolus of IV corticosteroids and AZA. The patient was treated with sectorial panphotocoagulation (PRP) laser, leading to the quiescence of ophthalmic involvement.

### 4.4 Mild lupus retinopathy to severe vaso-occlusive disease

#### 4.4.1 Case number 4

A 40-year-old female patient presented with recent blurry vision in her right eye (BCVA was 20/25). Examination of the fundus found bilateral cotton wool spots at the posterior pole, predominantly in the right eye. The retinal fluorescein angiography (FA) showed retinal ischemia with multifocal areas of non-capillary perfusion at the early phase corresponding to Purtscher flecken. The intermediate phase of FA highlighted the presence of bilateral retinal arteriolar and capillary occlusions. The indocyanine green angiography (ICG) found areas of hypofluorescence corresponding to the masking effect of cotton wool spots and Purtcher flecken without choroidal ischemia. The assessment in IM led to the patient being diagnosed with SLE with positive ANA and positive anti-SSA antibodies. No anti-phospholipid or thrombophilia factors were found. The general picture remained peculiar, owing to the late onset of SLE and initial necrotizing vascular involvement of the two lower limbs. The patient was treated with IV infusions of methylprednisolone and relayed by oral prednisone along with IV rituximab. Given that the patient’s retinal features were unchanged after this, treatment was reinforced with the introduction of oral CYC and PRP.

#### 4.4.2 Case number 5

A 46-year-old female patient of Vietnamese origin with primary Gougerot–Sjögren syndrome with no systemic complications visited the emergency room (ER) with symptoms of fever, polyadenopathy, and decreased visual acuity. Her previous work-up showed positive ANA titers at 1/1280 along with positive anti-SSA and SSB antibodies. She presented with a known history of dry skin and oral manifestations of Sjogren syndrome (xerostomia). The biopsy of the accessory salivary glands had a result of Chisholm III. SLE was diagnosed 10 years prior to the current episode because of symptoms of polyarthralgia, erythema of the face, and alopecia. The SLE had been treated with oral HCQ until 3 years ago. Upon presentation of the ocular symptoms, pseudo-retinopathy of Purtscher with bilateral major retinal ischemia and left macular ischemia were diagnosed. Her examination found that BCVA was corrected to 20/20 in the right eye and counting fingers (CF) in the left eye. Fundus examination showed multiple cotton wool spots as well as Purtscher flecken without hemorrhages, predominantly in the left eye. Treatment with a panretinal photocoagulation (PRP) laser was quickly initiated. Assessment by an IM specialist found multiple sub and under diaphragmatic lymphadenopathies that were hypermetabolic on PET scan assessment. The lab work found normocytic anemia with neutropenia, positive speckled ANA titers at 1/1280, complementary consumption of soluble nuclear antigen (ENA) positive antibodies, and anti-Sm, SSA-Ro, and SSB-La antibodies. Circulating anticoagulant antibodies were not detected. Lymphocytic meningitis with hyperproteinorachia was diagnosed associated by major hypergammaglobulinemia. An absence of APL syndrome was noted. The initial treatment was with corticosteroid infusions, followed by oral prednisolone with slow taper and IV pulses of CYC (Eurolupus protocol) and treatment with IV rituximab infusions.

#### 4.4.3 Case number 6

A 23-year-old female patient was referred for acute major bilateral visual loss. The BCVA was 20/200 in the right eye and 20/500 in the left. Three months earlier, the patient reported an influenza-like illness accompanied by anemia, biological inflammatory syndrome, disturbances in liver function, and a rash suggestive of SLE. The ophthalmic examination found a pseudo-retinopathy of Purtscher flecken presenting as a bilateral ischemic retinopathy. It presented as severe retinal arterial ischemia with ghost arteries in the four retinal quadrants, veins of irregular caliber accompanied by cotton wool spots or Purtscher flecken to the posterior pole, flame-shaped retinal hemorrhages, and vascular sheathing. FA confirmed ischemic retinal vasculitis predominantly in the left eye. Retinal PRP laser treatment of the left eye was quickly initiated. The assessment by IM found ANA levels to be greater than 1/1280, and the presence of negative anti-dsDNA, positive anti-Sm antibodies, and positive anti-RNP antibodies. There was no evidence of antiphospholipid syndrome. The patient received an emergent treatment with IV corticosteroid associated with CYC to control ischemic ophthalmic involvement.

#### 4.4.4 Case number 7

**Figure 3:**
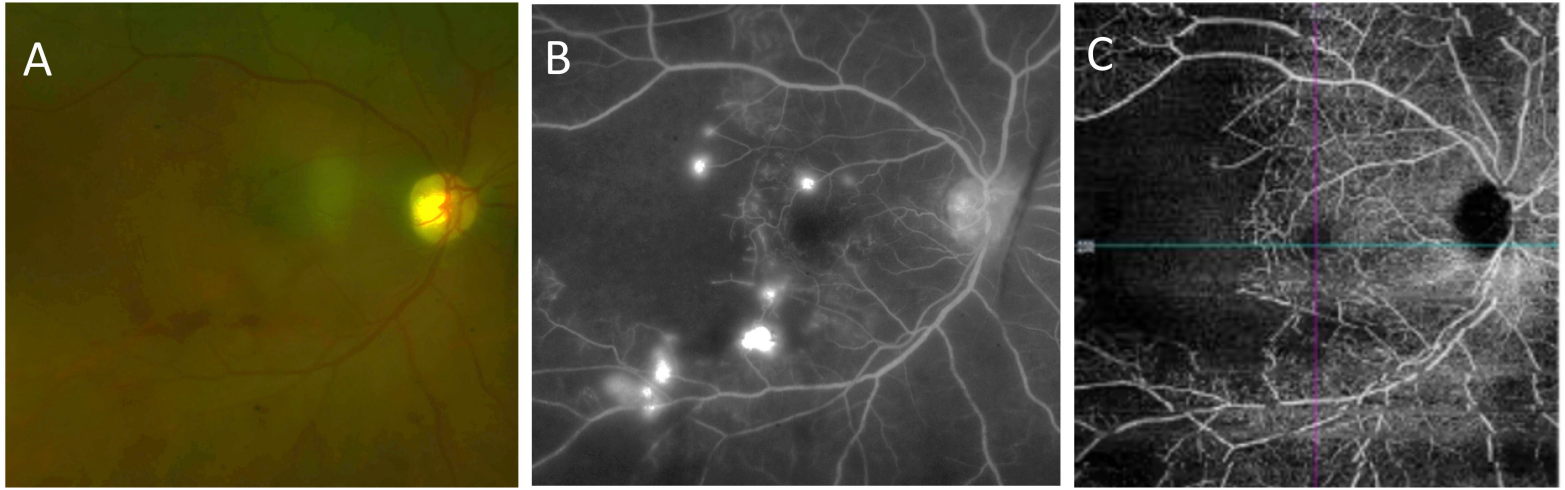
Case number 7. Multimodal imaging of a 48-year-old female with polyarthralgias, diffuse alopecia, ulceration of the hard palate, dyspnea, proteinuria subsequently diagnosed as class-III lupus nephritis, hypergammaglobulinemia and positive anti-Smith antibodies that presented to the ED with acute onset vision loss of the right eye following a biopsy of an axillary lymph node that demonstrated mixed lympho-plasmocytic proliferation compatible with florid systemic lupus. She had been on IV CYC, and oral MMF prior to the ophthalmic manifestations. **(A)** Fundus photograph shows macular ischemia with inferior hemorrhage and pale looking retina. **(B)** Late phase FA shows multiple pinpoint areas of leakage with a large area of nonperfusion temporally. **(C)** OCTA clearly shows lack of blood flow temporal to the fovea. Due the severe retinal ischemia the patient was treated with 1000 mg IV methylprednisolone initially, multiple intravitreal triamcinolone and anti-VEGF injections were needed shortly thereafter due to recurrent vitreous hemorrhages, and the addition hydroxychloroquine was required for further control of intraocular signs of active disease.

A 48-year-old female was evaluated for SLE following symptoms of malar rash, inflammatory arthritis, diffuse alopecia, nonpainful ulceration on the hard palate, dyspnea, and proteinuria. ANA was found to be positive and high titer, 1/1280 speckled pattern. She was also found to have high titers of anti-Smith antibodies, anti-SSA antibodies, low complement levels, hypergammaglobulinemia, and a high ESR of 120 and CRP of 11.1. At this time, the renal biopsy showed the presence of mesangial proliferative lupus nephritis, class II. Electron microscopy revealed multiple small to medium-sized subendothelial and subepithelial immune complex deposits, involving several capillary loops, suggesting a possible early transformation to class III-A lupus nephritis. On immunofluorescence, there was granular full-house Ig staining. Treatment with IV CYC 500-mg infusions was administered every 2 weeks for 3 months, followed by maintenance with oral MMF 1,000 mg twice per day, which then decreased to 1,500 mg daily. Shortly after the initial diagnosis of SLE, the patient presented to the ED with acute loss of vision in her right eye. The retinal fluorescein angiography was consistent with a large area of capillary non-perfusion with arteriolar involvement and telangiectatic vessels with new vessel leakage at the border of retinal ischemia (**Figure 3**). At that time, the patient received 1,000 mg of IV methylprednisolone for 3 days. Concurrently, enlarged right axillary lymph node was detected on physical examination. The axillary node biopsy demonstrated mixed lymphoplasmacytic proliferation. Per the pathology report, changes were potentially related to florid lupus erythematous. She also presented with a significant active cutaneous inflammation of the head and neck. The steroid treatment was followed by a marked improvement in the facial rash, but an ongoing blurriness in the right medial visual field secondary to the area of capillary drop out temporal from macula remained unchanged from time of discharge. Ongoing issues with retinal vasculitis, complicated by recurrent vitreous hemorrhages, occurred periodically over the next 5 years. Treatment included periocular triamcinolone injections, intravitreal anti-VEGF injections, 200 mg of oral HCQ twice per day, and topical prednisolone acetate 1%, and MMF was increased to 1000 mg twice per day owing to the possibility that her vitreous eye hemorrhages were related to ongoing inflammation from her SLE. The patient also had a history of recent active episcleritis in her left eye. A fundus examination of the right eye showed telangiectatic vessels at the macula. OCT angiography (OCTA) demonstrated an area of choroidal and retinal vasculature drop-out in the posterior pole.

### 4.5 Proliferative retinopathies

#### 4.5.1 Case number 8

**Figure 4:**
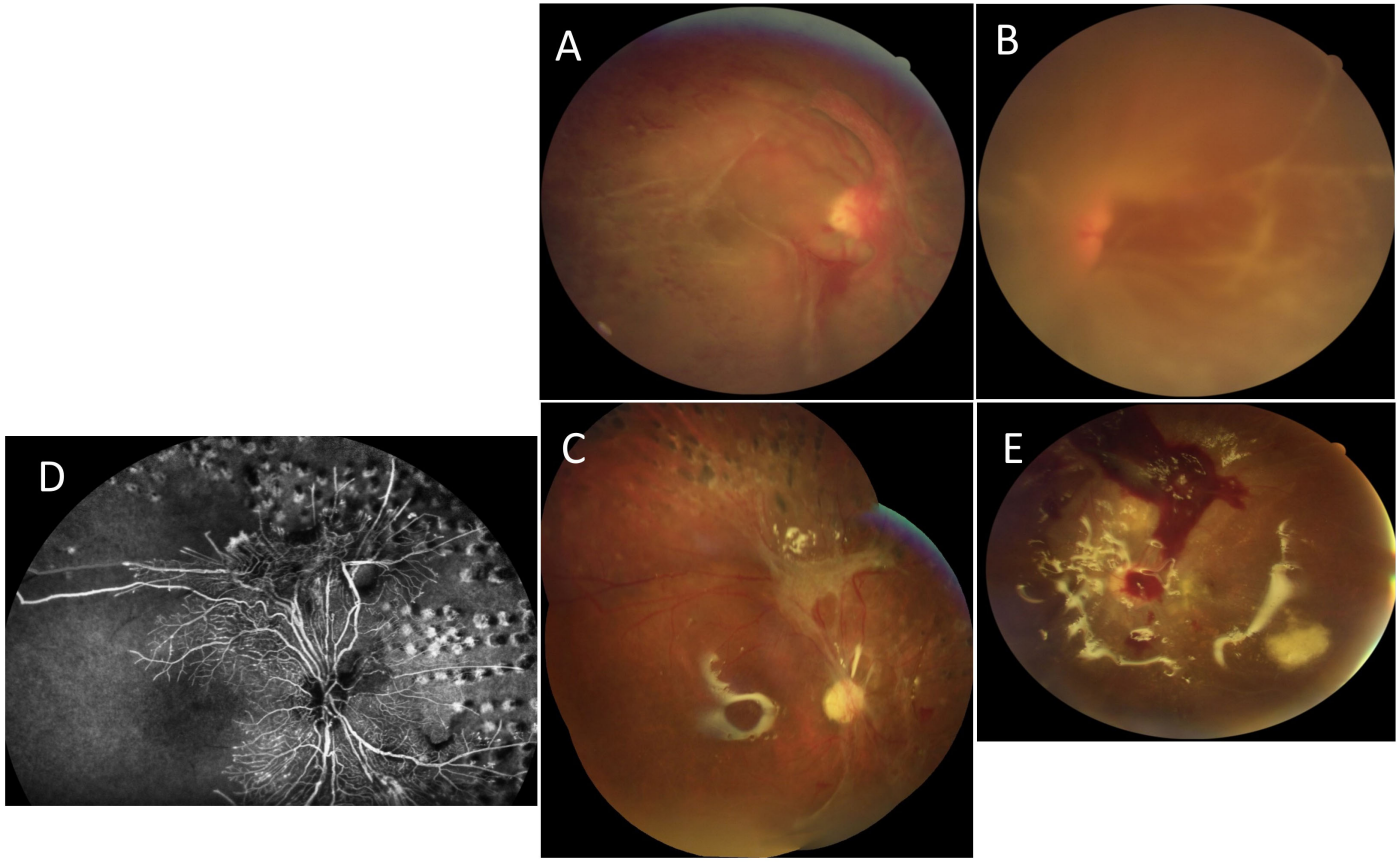
Case number 8. Multimodal imaging of a 20-year-old female from the Middle East with a diagnosis of mixed connective tissue disease following positive anti-dSDNA, anti-Smith, anti-RNP and anti-nucleosome antibodies and clinical signs that included oral ulcerations, polyarthralgia, malar rash squamous erythema of the ears and proteinuria diagnosed as extra-membranous lupus glomerulonephritis type V. She presented to the clinic with acute worsening of vision bilaterally. **(A)** Fundus photo of the right eye and **(B)** left eye show dense vitreous hemorrhage, vasculitis and tractional membranes. **(C)** Fundus photo of the right eye and **(E)** left eye following treatment with PPV, EL and SO. The left eye suffered from recurrent vitreous hemorrhage despite surgical interventions, which can be seen centrally and superiorly. **(D)** Wide field FA of the right eye with superior and central lack of perfusion, compatible with extensive macular ischemia. She was treated systemically with steroids, MMF and Rituximab.

A 20-year-old female patient of Kuwaiti origin, with no family history of ophthalmic issues, presented with a malar rash, oral ulcerations, inflammatory polyarthralgia, and biological inflammatory syndrome. She was brought to the ophthalmology department because of a recent worsening of visual acuity. The visual acuity showed that BCVA was reduced to hand motion (HM) in the right eye and LP in the left eye. A fundus examination showed the presence of vasculitis with severe ischemic retinopathy, complicated by tractional retinal detachment and bilateral vitreous hemorrhages (**Figure 4**). The patient was treated by bilateral pars plana vitrectomy with retinal traction delamination, retinal PRP laser, and silicone oil tamponade associated with intravitreal anti-VEGF injections. The clinical and laboratory investigations performed by IM showed inflammatory arthralgia, cervical lymphadenopathy, squamous erythema of the ears, thrombocytopenia, decreased haptoglobin, proteinuria at 3.03g/L, low CH50 at 60%, and normal sC3-C4. The biological assessment showed positive homogeneous, speckled ANA at > 1/1280, the presence of positive anti-dsDNA, positive anti-nucleosome antibodies, positive anti-Sm antibodies, and positive anti-RNP antibodies. The test for APL syndrome was positive for lupus anticoagulants, with negative aCL and negative anti-beta II GP1 antibodies. The renal puncture-biopsy revealed the presence of extra-membranous lupus glomerulonephritis, classified V (ISN/RPS). A systemic treatment was started with oral corticosteroids at 1 mg/kg, oral MMF at a dose of 2,000 grams per day and IV rituximab at a dose of 1 gram every 3 months.

#### 4.5.2 Case number 9

A 45-year-old female patient with known SLE who had required treatment with HCQ 20 years earlier was diagnosed with a vascular occlusion of the right eye. Systemic manifestations of lupus included cutaneous, articular, central nervous system involvement (CNS), and nephritic syndromes. Treatment for the ocular symptoms consisted of an intravitreal anti-VEGF injection, a sectoral retinal photocoagulation session, and oral corticosteroid therapy combined with MMF.

#### 4.5.3 Case number 10

A 59-year-old female patient with a 10-year medical history of SLE, characterized by cutaneous manifestations and nephritic syndrome, presented with ischemic retinal vasculitis complicated with bilateral retinal neovascularization. She received sectorial retinal laser PRP treatment. Systemic lupus and retinopathy remained inactive on a regimen of oral corticosteroids, oral AZA, and oral CYC over a follow-up period of 2 years.

### 4.6 Idiopathic intracranial hypertension

#### 4.6.1 Case number 11

**Figure 5:**
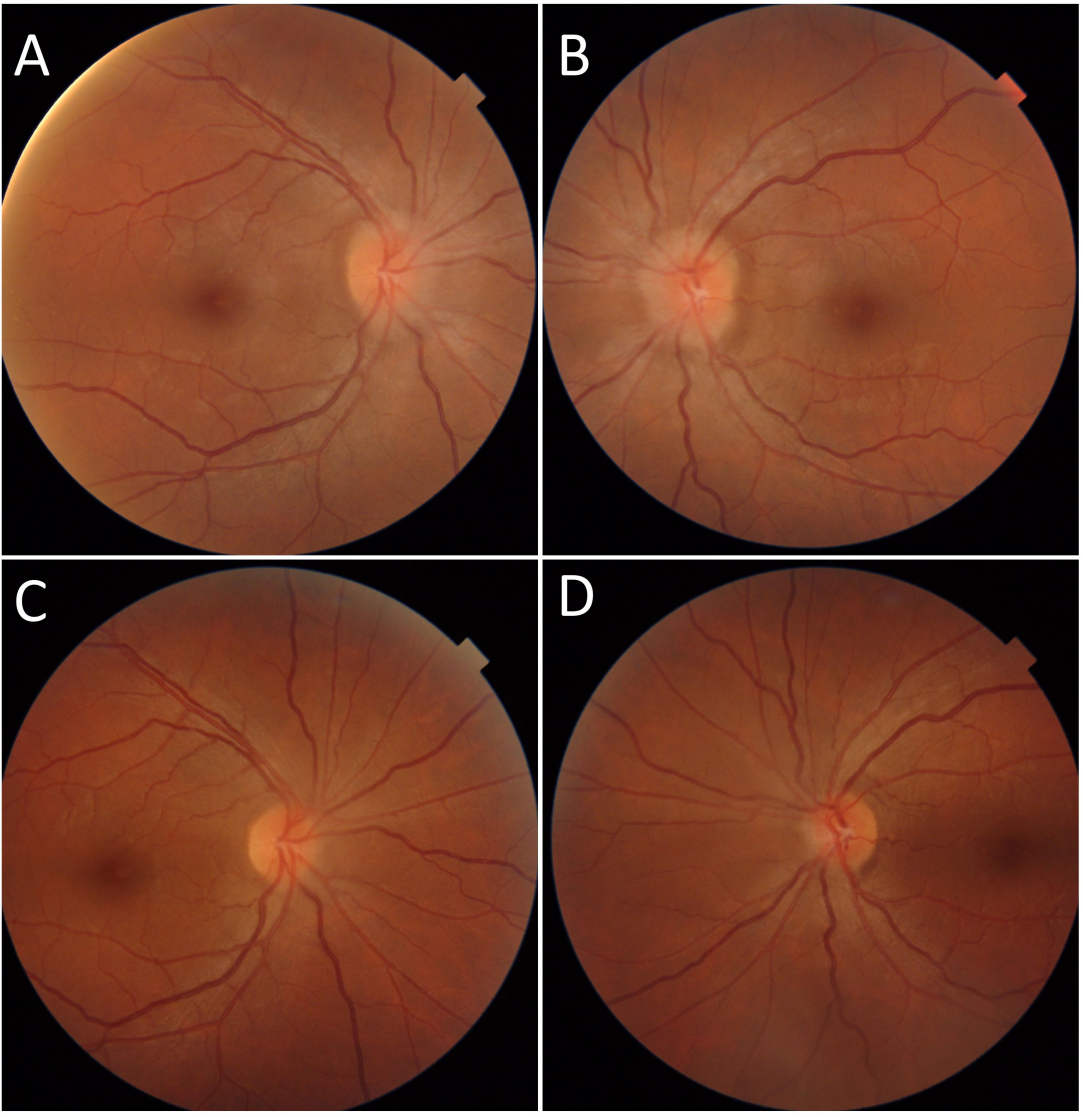
Case number 11. Fundus photographs of a 39-year-old female with SLE and chronic hydroxychloroquine use for 14 years. **(A)** There is Frisen grade 1 papilledema of the right eye and **(B)** Frisen grade 2 papilledema of the left eye. A negative MRI and increased cranial pressure yielded the diagnosis if IIH. Papilledema resolved following treatment with oral acetazolamide **(C, D)**.

A 39-year-old female patient with a history of SLE that had been treated for the past 14 years with HCQ (600 mg daily) and oral dapsone presented with asymptomatic bilateral papillary edema (**Figure 5**). She was seen in the ophthalmology department to receive her annual HCQ screening. She also had an ocular history of several granulomatous anterior uveitis flares. The assessment in IM with lumbar puncture and measurement of intracranial pressure led to a diagnosis of idiopathic intracranial hypertension (IIH). Oral acetazolamide treatment led to the reduction of papillary edema. The SLE manifested as subacute lupus associated with neutrophilic urticaria with polyarthralgias, biopsied polyadenopathies, and Sjögren’s syndrome, with dry mouth but without dry eyes. The laboratory results were significant, showing ANA > 1/1280, and the presence of positive anti-SSA and mixed type 2 cryoglobulinemia with monoclonal Kappa IgG. The test for APL syndrome was negative.

### 4.7 Posterior scleritis

#### 4.7.1 Case number 12

**Figure 6:**
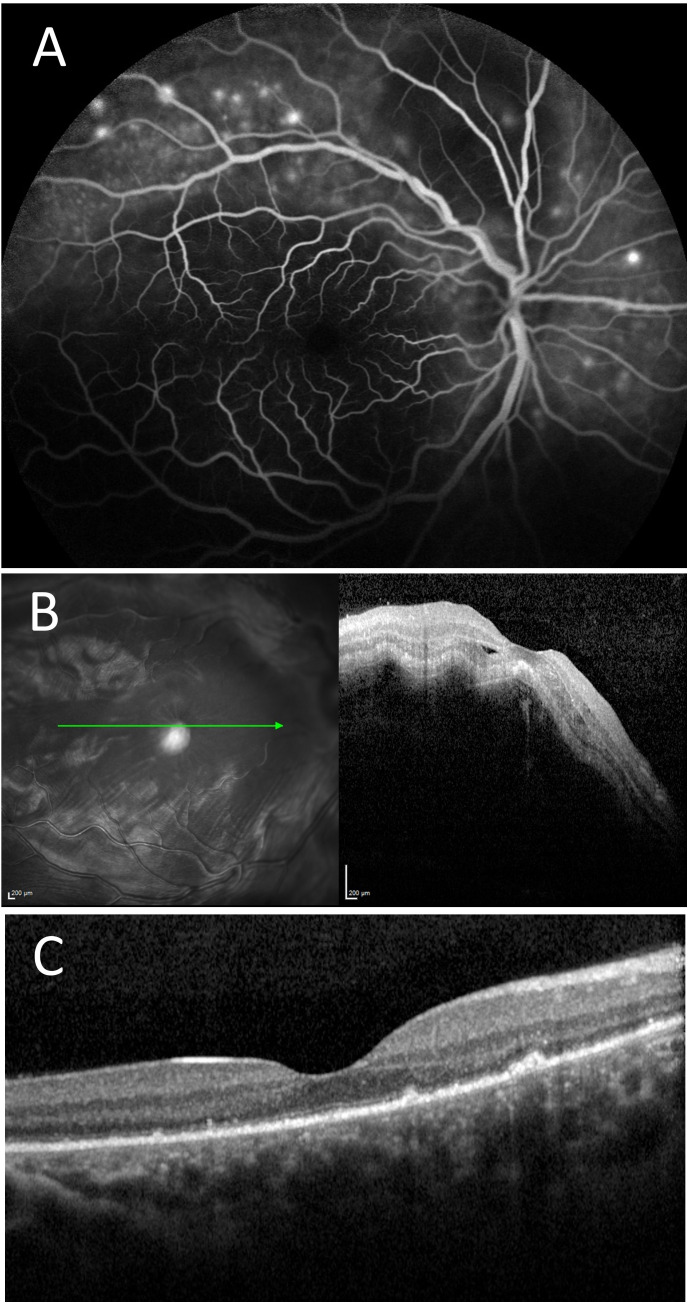
Case number 12. Multimodal imaging of a 37-year-old woman with obstetric and thrombotic antiphospholipid syndrome, and clinical signs cutaneous-articular lupus associated with pulmonary embolism quiet on oral 7.5mg of prednisone. She initially complained of decreased visual acuity of the left eye following a period of severe anxiety. **(A)** FA of the right eye shows multiple pinpoint areas of staining and a central hypofluorescent area compatible with a serous detachment. **(B)** OCT show thickened serous detachment compatible with posterior scleritis. Systemic mycophenolate mofetil allowed for the control of ophthalmic and systemic symptoms with resolution of serous detachment on SD-OCT **(C)**.

A 37-year-old female patient with a 16-year medical history of cutaneous-articular-nephritic lupus with obstetric and thrombotic APL syndrome complicated with pulmonary embolism presented to the ER. She had a history of intolerance to HCQ, but the symptoms of lupus were suppressed with a regimen of oral prednisone at 7.5 mg per day. In the ER, she complained of a reduced BCVA at 20/63 in the left eye in the context of severe anxiety. The fundus examination found a serous retinal detachment. A diagnosis of central serous chorioretinopathy after which the patient began treatment with corticosteroids. There was a good outcome with the taper of oral corticosteroids. Two months later, the patient experienced a decrease in BCVA of the right eye at 20/63 with a red eye with conjunctival hyperemia and chemosis. The fundus examination showed an exudative subretinal detachment with several hyperfluorescent pin points on FA (**Figure 6**). Posterior scleritis was confirmed by way of a B scan ultrasound and improved after corticosteroid infusions. The laboratory results were significant, showing high anti-dsDNA and low C3 and C4 levels. The previous APL antibodies results were not available. Systemic lupus was treated with MMF, which allowed for the stabilization of both ophthalmic and joint symptoms.

### 4.8 Optic neuritis/neuropathy

#### 4.8.1 Case number 13

**Figure 7:**
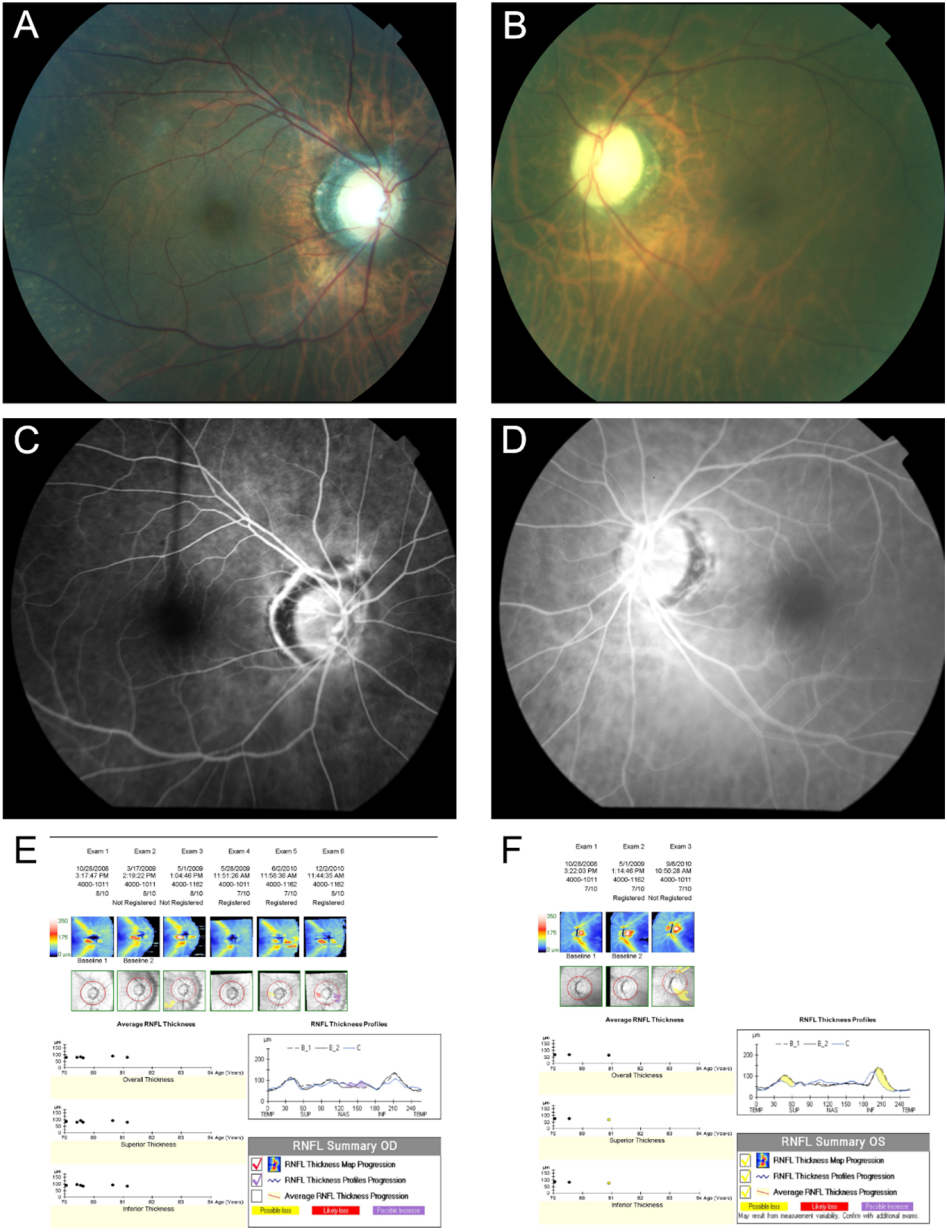
Case number 13. Multimodal imaging of a 77-year-old-female with a positive ANA, C3 and C4 SLE along with leukopenia. She presented with acute left eye vision loss and superior altitudinal defect despite being on oral prednisone. Fundus photo of the right **(A)** and Left eye **(B)** show a normal looking right optic nerve but a pale and atrophic left optic nerve. FA of the right **(C)** and **(D)** left eye show normal circulation without delays or leaks, ruling out giant cell arteritis or other vascular causes for the vision loss. The guided progression analysis (GPA) of the right **(E)** and left eye **(F)** shows progressive decline in the retinal nerve fiber layers of the left eye, compatible with atrophy.

A 77-year-old-female with a 10-year medical history of systemic lupus that manifested as leukopenia, borderline C3 and C4, and positive ANA presented for the evaluation of vision loss in the left eye and a worsening superior visual defect. Her medical history included Sjögren’s syndrome and transverse myelitis secondary to the SLE/Sjögren’s syndrome overlap. She suffered from an acute atraumatic subdural hemorrhage and a progressive vision loss in the left eye that promptly responded to systemic prednisone. This left her with a mild to moderate superior scotoma that remained stable for 7 years. When she presented to us, she was taking 7.5 mg of prednisone per day. Her initial BCVA was 20/100 in the left eye, without an afferent pupillary defect (APD) and dyschromatopsia. The results of the fundus examination were consistent with a pale left optic nerve and normal fluorescein angiography (**Figure 7**). She was admitted for IV solumedrol and MRI Brain and Orbits, which revealed optic nerve enhancement consistent with inflammatory optic neuritis which was responsive to steroids. MMF was started. The BCVA at last follow-up was 20/400 in the left eye.

## 5 Discussion

According to the literature, keratoconjunctivitis sicca is the most frequent ophthalmic manifestation of SLE (14), but it was reported in the patient’s notes in our series in only two cases (15%). The most prevalent ophthalmological clinical feature was lupus retinopathy, with varying degrees of severity. Vessel occlusions were mainly arteriolar or found in larger arteries or veins. A few other ophthalmological disorders were present, such as one eye presenting with papilledema revealing idiopathic intracranial hypertension, which is a well-known SLE association (15), another eye with posterior scleritis, along with anterior involvement such as anterior uveitis, keratoconjunctivitis sicca, periorbital edema, scleritis/episcleritis, optic neuritis, and orbital involvement with inflammatory pseudo-tumor orbital mass. Clinical manifestations were heterogeneous, as were visual acuity recoveries. Five out of nine eyes with an initial BCVA of ≤ 20/63 showed an improvement in BCVA after treatment. Because SLE is a severe disease, particularly as it can lead to renal involvement, a prompt diagnosis is necessary for initiating the appropriate therapy. We show here that ocular involvement may help with early detection of SLE as the diagnosis of ocular lupus was concomitant to the systemic lupus symptoms in our series in three out of 13 cases (23%) (case numbers: 4, 6, and 8). According to the literature, ophthalmic manifestations can be detected in approximately one-third of patients (14). Given the prevalence of retinal vasculitis/vaso-occlusive disease in particular, there is a possibility that SLE is underdiagnosed in patients with ocular manifestations who do not get the proper work-up as per ACR criteria. Prompt diagnosis through referral to internal medicine or rheumatology departments for uveitis work-up and treatment of the ophthalmic symptoms is important to preserve vision and to check for systemic manifestations of SLE.

We have not shown in our case series other ophthalmological manifestations, such as eyelids affected with plaques, erythematosus patches and madarosis, orbital inflammatory syndrome and myositis, and corneal involvement such as PUK.

Data from the literature indicate that the diagnosis of SLE is challenging. The 1982 revised ACR SLE classification criteria (16) and their 1997 revision (13) have been used worldwide. Although not part of the ACR’s lupus diagnostic criteria, ophthalmic manifestations have been included in the BILAG criteria (16) to characterize the severity of lupus disease (11).

There are other important ocular manifestations of active SLE that are not considered in the BILAG criteria, which score activity in the last month across nine domains (constitutional, musculoskeletal, mucocutaneous, hematological, renal, abdominal, ophthalmic, cardiorespiratory, and abdominal). For example, intracranial hypertension has been reported in association with severe SLE and lupus nephritis (17), and may be associated with sight-threatening papilledema, as stated by Jawahar et al. (18). Our current series is in accordance with their findings because we present herein a patient with bilateral papilledema who had a 14-year history of SLE treated by HCQ and who it was discovered suffered from IIH.

A recent 2021 systemic review involved a systematic literature search to identify cohort, case–control, and cross-sectional studies about the epidemiology of ophthalmological manifestations in active SLE. It showed that the prevalence of each of the ocular manifestations related to SLE, with the exception of retinal vaso-occlusive disease, was consistently less than 5% (18). The prevalence of episcleritis ranged from 0% to 4.2% in the studies referenced by Jawahar et al. in their systemic literature search (19–22). They found that the prevalence of peripheral ulcerative keratitis was 0.56% and 4.1% in two studies of 17,942 and 9,843 patients, respectively (23, 24); the prevalence of anterior uveitis was 0.35% (23, 25); and the prevalence of retinal vasculitis without vascular occlusion was 0%–2.9% across four studies (19, 26–28). The prevalence of optic neuritis ranged around 1% across studies in the SLE population (29, 30). Finally, Jawahar et al.’s literature search found that the prevalence of anterior ischemic optic neuropathy ranged from 0% to 1.22% across two studies rated as being at medium risk of bias (24, 31). The prevalence of the retinal vaso-occlusive disease form of ocular lupus ranged from 0% to 7.31% across eight studies (19, 23, 26–28, 31–34).

Ophthalmological involvement of SLE, although rare, can be very functionally severe with the presence of retinal ischemia. In our current cohort of patients with recent onset ocular SLE, the majority of patients (eight out of 13) presented with occlusive lupus retinopathy. The pathophysiology of Purtcher pseudo-retinopathy is still uncertain. A plausible hypothesis is that of microembolizations with pre-capillary arteriolar occlusions and microvascular infarctions of the retinal nerve fiber layer (35). This microembolization could be due to the aggregation of lymphocytes. Possible endothelial abnormalities and hyperviscosity would promote these microembolizations. Both the T and B cells of SLE patients display multiple signaling abnormalities, leading to intrinsic hyperactivity and hyper-responsiveness of T and B cells (36). This microcirculatory vascular part must also discuss an associated APL syndrome that must be excluded systematically. For the treatment of lupus retinopathy, immunosuppressants and/or anticoagulant therapy may be advised. High-dose steroids should be used in patients with severe ocular manifestations as this is typically associated with high disease activity (37).

According to the European League Against Rheumatism (EULAR), the first-line treatment of systemic lupus without major organ involvement includes HCQ, low-dose glucocorticoids, or NSAIDs. Additional immunosuppressive drugs such as methotrexate, AZA, CYC, and MMF are recommended for those patients who do not respond to the initial treatment (38).

Antimalarials such as HCQ and chloroquine are often included as the first-line treatment of lupus because of their ability to treat the constitutional symptoms of lupus along with the musculoskeletal and cutaneous manifestations (39). However, antimalarials have also been associated with severe retinal toxicity, requiring a discontinuation of therapy. Although this toxicity is rare, the incidence increases with duration of treatment, exceeding 1% after 5–7 years (40).

Along with HCQ, treatment of lupus involves immunosuppression through medications such as methotrexate, AZA, CYC, and MMF. However, there are no guidelines in relation to the treatment of ophthalmic lupus. The methotrexate trial had only one patient with unspecified ophthalmic disease in each of the case and control arms, whereas the neuropsychatric lupus CYC study had one patient with optic neuritis in the control arm and three patients in the treatment arm, which was insufficient to conduct an ophthalmic subgroup analysis. Disease-modifying antirheumatic drugs (DMARDs), MMF (2 and 3 g total daily doses), or IV CYC along with glucocorticoids are suggested as induction therapy for lupus nephritis classes III and IV. For CYC, there are two regimens of IV doses (1): low-dose “Eurolupus” CYC (500 mg IV once every 2 weeks for a total of six doses) followed by maintenance therapy with daily oral AZA or daily oral MMF (Level B) and (2) high-dose CYC (500–1,000 mg/M2 IV once per month for 6 doses) followed by maintenance treatment with MMF or AZA. There is no consensus regarding the use of calcineurin inhibitors (cyclosporine, tacrolimus, voclosporin); however, there is evidence for their efficacy as induction agents and in refractory disease. AZA is not recommended as one of the first choices for induction therapy (41).

Current treatments of systemic lupus are shifting toward more targeted immunosuppression. B cells have been found to have a central role in the pathogenesis of lupus; therefore, B-cell-targeted therapies, including those targeting B-cell surface antigens (rituximab, obinutuzumab, epratuzumab), B-cell survival factors (belimumab, tabalumab, blisibimod), or B-cell intracellular functions (proteasome inhibitors) are being used for treating SLE (42).

The American College of Rheumatology guidelines for treatment of lupus nephritis in patients who fail to respond after 6 months of treatment with glucocorticoids plus MMF or CYC recommends the switching of the immunosuppressive agent from CYC to MMF, from MMF to CYC, or from either to rituximab (41).Two randomized controlled trials were conducted to evaluate the use of rituximab to treat SLE: the Exploratory Phase II/III SLE Evaluation of Rituximab (EXPLORER) trial and the Lupus Nephritis Assessment with Rituximab (LUNAR) trial, both of which failed to meet their primary end point for treatment of extrarenal and lupus nephritis. However, the EXPLORER trial showed reduced risk and frequency of SLE flare (43) and the LUNAR trial showed normalized complement levels, proteinuria, and anti-dsDNA autoantibody levels (44). Rituximab is not approved by the FDA in the United States to treat systemic lupus, but the use of rituximab is supported as one option for the treatment of refractory lupus in the EULAR and ACR guidelines (45, 46).

There has been a number of case reports on the positive response of rituximab in ophthalmic complications of SLE, in particular, SLE retinal vasculitis. Rituximab has been used in combination with MMF and oral prednisolone after plasma exchange (47) or with CYC and oral corticosteroids (48) to treat retinal vasculitis. Similarly, Dhirani et al. showed the efficacy of plasmapheresis therapy (five sessions over a 10-day span), bilateral administration of intravitreal ranibizumab (0.5 mg), and IV rituximab therapy post plasmapheresis in a case of lupus retinal vasculitis refractory to IV high-dose pulse steroid treatment (49). They hypothesized that the combined effect of their therapy was able to provide a significant and immediate reduction of intravascular inflammatory complexes in the arterioles and reduce antibody mediated inflammation of the vessel wall leading to retinal damage (49–51).

A few other anti-CD20 and anti-CD22 monoclonal antibodies are also being investigated for their potential use in the treatment of SLE. Obinutuzumab, a humanized type II anti-CD20 monoclonal antibody, has shown improved renal responses in patients with lupus nephritis (52). Additionally, Epratuzamab, an anti-CD22 monoclonal IgG antibody, has been shown to be effective in moderate/severe active SLE, with phase III studies (the EMBODY studies) showing improvement in disease activity and bioactivity. However, patients with active ophthalmic disease have not been recruited (53).

Other B-cell-targeted therapies show great promise in playing a role in SLE therapy but have so far not shown efficacy in the treatment of ophthalmic lupus. Belimumab is a human monoclonal antibody that targets B-cell activating factor (BAFF) inhibitors. Belimumab was approved for treatment of seropositive, moderate SLE. Data are currently lacking for a role of belimumab in nonrenal lupus requiring CYC (54). As stated by Papagiannuli et al. in their systematic review of the literature, no patients with ocular disease were included in these trials, and for the moment it seems unlikely that belimumab will have a role to play in managing the ophthalmic complications of SLE (55).

Similar to belimumab, tabalumab and blisibimod are B-cell activating factor (BAFF) inhibitors and they have shown promising results in phase III trials, although the trial end points were not met. Blisibimod was associated with successful steroid reduction, decreased proteinuria, and biomarker responses (56), and tabalumab showed significant reductions in anti-dsDNA antibodies, increases in C3 and C4, and reductions in total levels of B cells and immunoglobulins (57). Ocular lupus was not listed among the SLE characteristics in these trials.

The proteasome inhibitor bortezomib showed favorable therapeutic effects when used in combination with corticosteroids in patients with severe SLE manifestations irresponsive to conventional immunosuppressive agents (58). However, it also caused many adverse reactions in patients (59). Data about patients with ocular lupus are also missing in these studies.

Blood interferon alpha concentrations are known to increase with disease activity in patients with systemic lupus. Thus, anti-IFN-alpha antibodies such as rontalizumab and sifalimumab, along with anti-type 1 IFN receptor antibodies (anifrolumab), and a vaccine preparation inducing anti-IFN-α antibodies (IFN- α kinoid), have been developed (60). Anifrolumab is a human monoclonal antibody that binds to the type I interferon receptor subunit 1, which prevents type 1 IFN-mediated signaling (61) and has been approved by the FDA for patients with systemic lupus. Although there have not been studies showing its effectiveness in ocular lupus, in the TULIP-1 study there was one patient with ophthalmic lupus included, and no flares were noted after treatment with anifrolumab in this patient (62).

Other therapies being explored are JAK inhibitors, tyrosine kinases that are involved in the intracellular signaling transduction (38, 63). Phase 1 trials of tofacitinib show some clinical responses (38). A case study showed that this drug could be useful for a patient with periorbital edema associated with lupus who did not respond to treatment with tacrolimus and prednisone (64).

The management of ophthalmic manifestations of active SLE can also include treatments such as photocoagulation or anti-VEGF therapy to prevent neovascularization in retinal vascular occlusive disease, vitrectomy for cases involving vitreous hemorrhaging, scleral buckling for retinal detachment, acetazolamide for elevated intraocular pressure, and dexamethasone implants for macular edema (65). The treatment of keratoconjunctivitis sicca is related to its severity. Artificial tear drops, cyclosporine-A (CsA) ophthalmic emulsion 0.05%, steroid eye drops, punctal plugs, and autologous serum eye drops have been used (66).

We can conclude that the treatment of ocular lupus may differ between renal and nonrenal lupus treatment and, therefore, it is important to take this into account. Although ophthalmic manifestations of active SLE are uncommon and not part of the ACR’s lupus diagnostic criteria, because of their functional severity, they have been included in the BILAG criteria (16) for determining the severity of lupus disease. As shown in our case study series, the ocular localization of the pathology may lead to the diagnosis of SLE, which is a complex autoimmune disease with heterogenous clinical manifestations. A recent observational study showed that the diagnosis of SLE is delayed by more than 6 months in three-quarters of patients (67). Therefore, it is of critical importance that ophthalmologists are able to recognize the ophthalmic manifestations of lupus and obtain critical work-up to detect systemic lupus earlier and thereby improve outcomes.

## Data availability statement

The raw data supporting the conclusions of this article will be made available by the authors, without undue reservation.

## Ethics statement

The studies involving human participants were reviewed and approved by STUDY19010072. Written informed consent for participation was not required for this study in accordance with the national legislation and the institutional requirements. Written informed consent was obtained from the individual(s) for the publication of any potentially identifiable images or data included in this article.

## Author contributions

Writing the original draft and preparing edits: NK, M-HE, MP-E, and VT; Editing: DS, NK, M-HE, MP-E, and VT; Supervising the paper and editing the first draft: DG, RA, AQ-K, FA, CB, GC, VC, MS, NS, EH, P-AB, BB, SM-U, and A-JS. All authors contributed to the article and approved the submitted version.
